# The role of the paravertebral muscles in adolescent idiopathic scoliosis evaluated by temporary paralysis

**DOI:** 10.1186/s13013-017-0138-7

**Published:** 2017-10-10

**Authors:** Christian Wong, Kasper Gosvig, Stig Sonne-Holm

**Affiliations:** 1 Department of Orthopaedics, University Hospital of Hvidovre, Kettegaard Allé 30, 2650 Hvidovre, Denmark; 2 Department of Radiology, University Hospital of Hvidovre, Kettegaard Allé 30, 2650 Hvidovre, Denmark

**Keywords:** Injection therapy, Botulinum toxin A, Idiopathic scoliosis, Prospective study, Radiological Cobb’s angle

## Abstract

**Background:**

Muscle imbalance has been suggested as implicated in the pathology of adolescent idiopathic scoliosis (AIS). The specific “pathomechanic” role of the paravertebral muscles as being scoliogenic (inducing scoliosis) or counteracting scoliosis in the initial development and maintenance of this spinal deformity has yet to be clarified in humans. In the present study, we investigated the radiographic changes of temporal paralysis using botulinum toxin A as localized injection therapy (ITB) in the psoas major muscle in AIS patients.

**Methods:**

Nine patients with AIS were injected one time with ITB using ultrasonic and EMG guidance in the selected spine muscles. Radiographic and clinical examinations were performed before and 6 weeks after the injection. Primary outcome parameters of radiological changes were analyzed using Wilcoxon signed-rank test and binomial test, and secondary outcome parameters of short- and long-term clinical effects were obtained.

**Results:**

Significant radiological corrective changes were seen in the frontal plane in the thoracic and lumbar spine as well as significant derotational corrective change in the lumbar spine according to Cobb’s angle measurements and to Nash and Moe’s classification, respectively. No serious adverse events were detected at follow-up.

**Conclusions:**

In conclusion, this study demonstrated that the psoas major muscle do play a role into the pathology in adolescent idiopathic scoliosis by maintaining the curvature of the lumbar spine and thoracic spine.

**Trial registration:**

EudraCT number 2008-004584-19

## Background

**Fig. 1:**
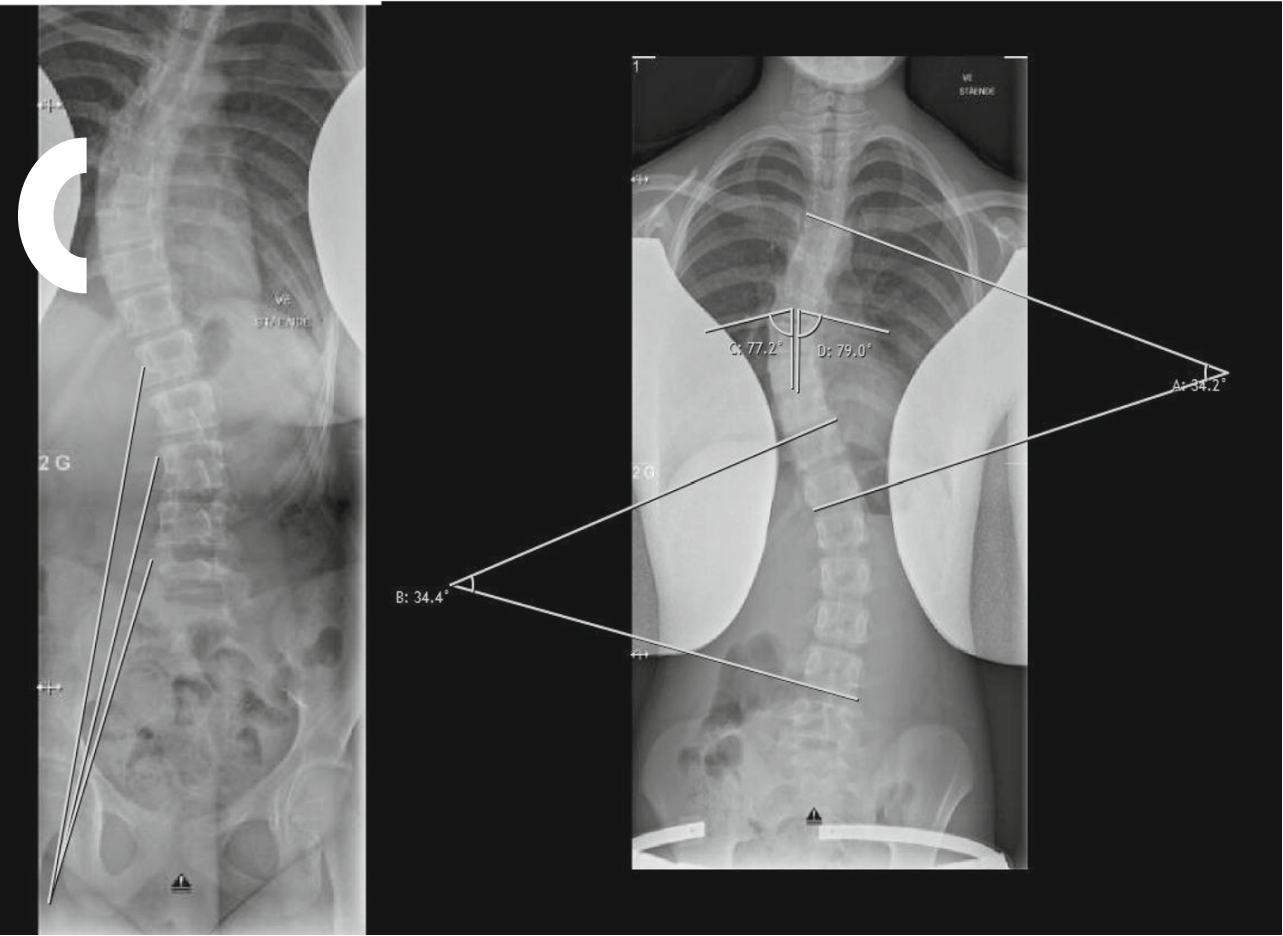
Schematic representation of the psoas major on the concave side of a thoracic scoliosis; C marks the stronger muscles on the thoracic convex side scoliosis in accordance with the literature (left). Measurements of thoracic and lumbar Cobb’s angle and concave and convex rib vertebra angle (right)

The Greek physician Hippocrates was the first to describe adolescent idiopathic scoliosis (AIS) as early as 400 BC [1]. Today, the etiology of AIS is still considered multifactorial, even though over time many researchers have tried to explain the pathology by one single etiology, ranging from a broad variety of causes of either biomechanical or genetic nature [2–6]. One relative recent observation by Modi et al. is that the spinal deformity in mild AIS tries to return to the neutral midline position, thereby displaying a “wavy” curve pattern with fluctuations in a lateral curve shape when followed closely [4]. They suggested that the paravertebral muscles would have a “tuning/balancing mechanism” that tries to correct the spinal deformity of mild scoliosis into apparent spontaneous regression or to prevent further progression of curve, and if failing, this would result in further progression [4]. The natural history of AIS, where the majority spontaneously remains stable while the rest either regresses or progresses, may be seen as suggestive for this hypothesis [7], and the paravertebral muscles or rather a misbalance of the paravertebral muscles has been suggested as causative for progression or regression of AIS [3, 4, 6, 8, 9]. Differences in morphology examined by MRI, behavioral response to exercise, and electromyographic response of the paravertebral muscle have indicated that muscle imbalance may play a role in the pathologic pathway that leads to progression or regression of AIS. This important question of the “pathomechanic role” of paravertebral muscles is still debated [10], but the evidence for the specific role as scoliogenic (inducing scoliosis) or counteracting scoliosis in human is still circumstantial [2, 3, 9–13]. Recently, Grivas et al. examined this for the quadratus lumborum muscle by comparing the length of the 12th rib in a group of children with right lumbar idiopathic scoliosis and straight spines; he suggested that stimulation of the paravertebral muscles should be performed to determine the “pathomechanic role” in future studies [10]. In this study, we examined if the hypnotized “pathomechanic role” of the psoas major (PM) of the iliopsoas muscle is scoliogenic—not by stimulation but by paralysis. The PM muscle is interesting for examining the “pathomechanic role” for AIS; Bruggi et al. found an interrelationship between the paravertebral muscle iliopsoas and AIS, where the muscle in isometric contraction had a corrective effect of the scoliotic curve [14]. In addition, a volumetric asymmetry of the PM has also been demonstrated in patients with degenerative AIS, where hypertrophy of 6.3% on the convex side was concluded to be associated with the scoliosis [12]. Yet, another study was unable to demonstrate that this difference had a significant effect in either the maximal voluntary isometric contraction force between healthy girls (161.4 *N*) and girls with scoliosis (144.3 *N*) or in the strength of the paravertebral muscle on either side of the scoliosis [15]. This interest in the PM in regard to AIS stems from the anatomy of the PM; it is a long fusiform muscle that is distributed on the lateral side of the lumbar spine from Th12 to L5, where it inserts on the transverse processes, the two adjacent vertebral bodies and their intervertebral discs. Moreover, it inserts from a series of tendinous arches extending across the bodies of the lumbar vertebrae. The PM then descends through the pelvic brim and passes beneath the ligamentum inguinale. It is finally attached to the trochanter minor of the femur. The function of PM is that of having an antigravity compensation, which also acts as a stabilizer of the lumbar lordosis in an upright posture [16]. The hypnotized scoliogenic role of the PM muscle would be that of initiating or maintaining a lumbar scoliotic curvature by muscle contraction. The PM would act by performing a lateral pull in the upper part of the lumbar spine into a concave scoliotic curvature, thus creating a convex thoracic curve in the thoracic and thoracolumbar scoliosis. This is illustrated in Fig. 1. The PM muscle would seem an ideal case in which to examine and clarify this specific scoliogenic effect, since it is of such a strength/magnitude/size that temporary paralysis would affect the scoliotic curves when recorded radiographically and at the same time would be attainable for safe percutaneous injection treatment.

Botulinum toxin A as a localized injection therapy (ITB) has been utilized to reduce spasticity and improve the motor dysfunction in cerebral palsy. ITB has already been examined for neuromuscular scoliosis by injection in the back muscles for treatment, where the corrective and clinical efficacy was examined [17, 18]. However, to our knowledge, ITB for AIS has not been investigated and would seem to be ideal for examining the role of the muscles in AIS, since it provides temporary muscle paralysis without long-term side effects or complications in therapeutic doses in otherwise healthy humans [19]. In this study, we conducted a small longitudinal prospective series using ITB for AIS, examining the radiological changes when treatment would have maximal paralytic effects (after 6 weeks). The purpose was to examine if ITB would induce a change in curvature in AIS. This could clarify if the spinal muscles play in the pathological process of the AIS, whether the spinal muscles would in fact induce the spinal deformity of AIS by the muscle forces/pull of the PM, thus having a scoliogenic effect, where regression of the AIS after paralysis would happen.

## Methods

In the present study, the patients were recruited patients from those already being treated for AIS at our hospital from the out-patient clinic. We carried out inclusion after oral and written informed consent. Inclusion criteria included a history of AIS, an age between 10 and 14 years, and a Cobb’s angle of at least 10°. Exclusion criteria were hypersensitivity or allergy to botulinum toxin A, ongoing infection at the injection sites, or prior ITB within the last 6 months. The patients are characterized clinically in Table 1.

**Table 1:** Patient characteristics

Pt^a^	1	2	3	4	5	6	7	8	9
Age^b^	14.5 (13.3)	14.0 (2.8)	13.5 (13)	11.9 (19)	10.4 (11)	8.44 (1)	12.5 (51)	11.7 (5.9)	14.6 (10.7)
Sex^c^	fem	fem	fem	fem	fem	male	fem	fem	fem
Risser	0	4	0	0	1	2	4	4	4
Mena^d^	0^f^	13.6	14.2	0^e^	11.6	0^e^	16	12.3	14.2
Type of scoliosis^g^	Right TLS	Right TS	Right TLS	Right TS	Left TLS	Right TLS	Right TLS	Right TLS	Right TLS
Med. C^d^	BP + ED^h^	SPH + CM^h^	–	–	–	BP	–	–	BP

^a^Patient ID

^b^Age when diagnosed in years (time of injection after diagnosed in month)

^c^Gender (*fem* female, *male* male)

^d^Age of menarche (years)

^e^Before menarche

^f^No menarche due to hormonal imbalance

^g^Type of scoliosis (*right* right-handed, *left* left-handed, *TLS* S-shaped convex thoracolumbar scoliosis, *TS* thoracic scoliosis)

^h^Medical condition before injection (*BP* back pain, *SPH* physiological disorder of schizophrenia, *ED* eating disorder of anorexia, *CM* cyst in medulla)

The injection treatment was given as a standard dose with three injections on the concave side of the lumbar scoliosis in the PM part of the iliopsoas muscle, so that the maximum dose in the single muscle did not exceed 100 units as in the earlier studies [17, 18]. After placement of the injection needle in the target muscle, we confirmed the correct placement by an ultrasound and by electric simulation through the needle for correct identification of the target muscle, since correct targeting of the deep back muscles otherwise seemed unreliable [20]. An experienced anesthesiologist and pediatric orthopedic surgeon performed the injections under general anesthesia using propofol infusion and spontaneous breathing, when lying in lateral position. We performed the radiographic examinations before and 6 weeks after injection treatment with standing radiographs, when botulinum toxin A would have the maximum effect on the muscles (visit window of 2 weeks). The same staff performed the radiographic acquisitions in a uniform, systematic manner, where patients omitted the brace for 24 h before the acquisition [21]. The primary outcome measures were the measurements of Cobb’s angle for primary and secondary curves, and the secondary parameters were the level of measurements for primary and secondary curves, rib vertebra angles for the thoracic apex vertebra, rib vertebrae angle difference, Nash and Moe’s classification at the apex vertebrae of the primary and secondary curves, and level of the apex vertebra for primary and secondary curves. Three experienced doctors performed all measurements similarly, separately and blinded, and we used the average results for further analyses. See Fig. 1 for a schematic representation of the radiographic evaluation.

Tertiary outcome measures were clinical, where patients and/or their parents were questioned openly at follow-up after treatment and specifically about their/the patient’s general well-being, about the effect of treatment in regard to brace tolerance if any, about respiratory problems, and about pain, endurance, and weight change. The statistical analyses performed on the study data were Wilcoxon signed-rank test (significance level 0.05) using SPSS (IBM Corp. released in 2013. IBM SPSS Statistics for Windows, Version 22.0. Armonk, NY: IBM Corp.) for measurements before and after ITB of Cobb’s angle (primary parameter), rib vertebra angle, and rib vertebrae angle difference and one sample binomial test for change in the levels of apex vertebrae and levels of curve measurements and Nash and Moe’s classification (significance level 0.05); if, in the Nash and Moe’s classification, the level of measurement of Cobb’s angle or apex vertebrae changed with one, we considered this as a change (+ 1), otherwise as no effect (0).

In this study, an *off label* medicine was used in children and adolescents. We obtained appropriate permissions from the Danish local ethical committee and the Danish Health and Medicine Authority (EudraCT number 2008-004584-19). The good clinical practice unit of Copenhagen monitored this study, and we screened the patients continuously for events, adverse events, and serious adverse events throughout the study period according to national guidelines, the European GCP guidelines, and the Helsinki II Declaration for biomedical research involving humans. We received no commercial or public financial support during the study.

## Results

Nine patients with AIS met the inclusion criteria. The patients maintained prior treatment of physiotherapy, bracing and otherwise throughout the study period. No patients were excluded, lost at follow-up, or withdrew from the study.

**Fig. 2:**
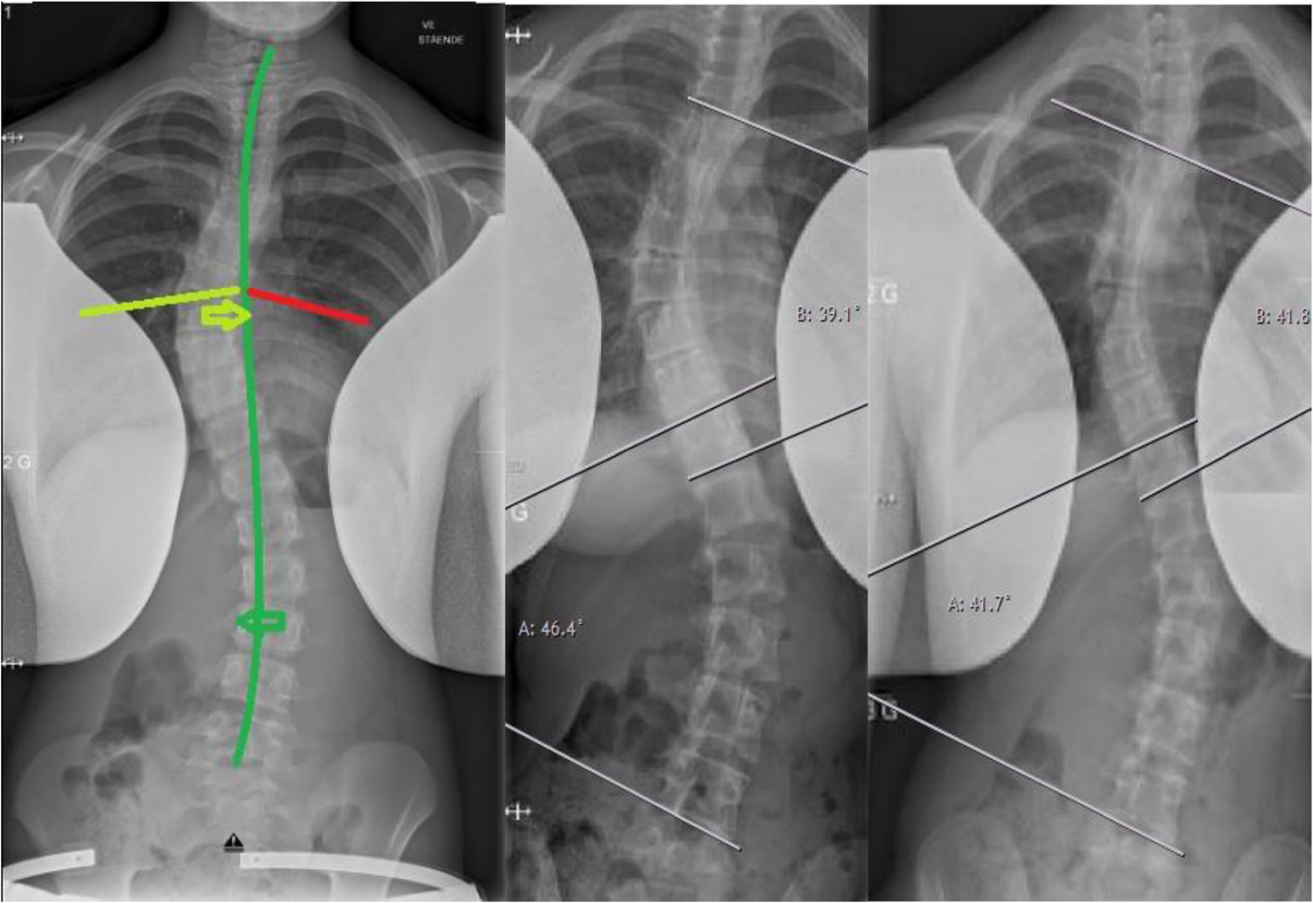
Changes in radiographic parameters. To the left: dark green, significant improvement in Cobb’s angle (curves) and Nash and Moe’s classification (error); light green, insignificant improvement in Nash and Moe’s classification (error) and in rib vertebra angle (line); red, insignificant deterioration in rib vertebra angle (line). To the middle and right: an example of pre- (middle) and post-injection (right) radiographs for patient number 9, where there is a smaller lumbar Cobb’s angle and larger thoracic Cobb’s angle after injection of botulinum toxin A

**Fig. 3:**
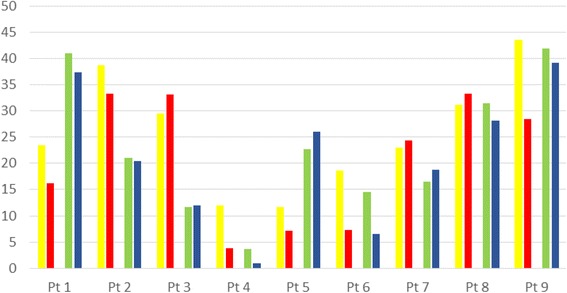
Changes in radiographic parameters: patient number on the ordinal axis (*x*) and Cobb’s angle on the vertical axis (*y*): yellow = Cobb’s angle pre-injection in the thoracic spine, red = Cobb’s angle post-injection in the thoracic spine, green = Cobb’s angle pre-injection in the lumbar spine, and blue = Cobb’s angle post-injection in the lumbar spine

The primary outcome parameters with their subsequent statistical analyses are shown in Table 2. Figures 2 and 3 illustrate the changes of radiographic parameters graphically and schematically.

**Table 2:** Radiological effects

Pt ID^a^	1	2	3	4	5	6	7	8	9	*P* value^b^
cobb t pre^c^	23.5(6.1)	38.7(8.1)	29.5(2.3)	12(19.6)	11.7(15.2)	18.6(14.0)	23(10.0)	31.1(17.0)	43.5(3.0)	
cobb t post^c^	16.2(12.2)	33.3(8.1)	33.1(3.3)	3.8(6.4)	7.1(4.5)	7.3(11.7)	24.4(16.4)	33.3(15.5)	28.4(3.0)	0.015**
cobb l pre^d^	40.9(7.3)	21(9.8)	11.6(13.8)	3.6(16.3)	22.7(9.6)	14.5(12.1)	16.5(12.0)	31.5(14.0)	41.8(10.4)	
cobb l post^d^	37.3(5.4)	20.4(0.1)	12(1.4)	1(2.6)	26(6.8)	6.5(3.6)	18.8(1.7)	28.2(18.3)	39.1(3.2)	0.038**
RA conc pre^e^	46.6	60.6	75.8	62.5	78.6	55.1	65.5	72.2	73.6	
RA conc post^e^	73	64	65.8	58.6	64.4	56.4	78.5	68.6	75	0.953
RA conv pre^f^	72.6	80.3	58.5	62.7	76.9	55.4	63.9	73.5	70.5	
RA conv post^f^	69.7	69.4	54.5	69.6	83.2	63.4	58.6	61.1	69.1	0.594
RVAD pre^g^	− 26	− 19.7	17.3	− 0.2	1.7	− 0.3	1.6	− 1.3	3.1	
RVAD post^g^	3.3	− 5.4	11.3	− 11	− 18.8	− 7	19.9	7.5	5.9	0.594
Pt ID^h^	Pre Pt1	Post Pt1	Pre Pt2	Post Pt2	Pre Pt3	Post Pt3	Pre Pt4	Post Pt4	Pre Pt5	Post Pt5
TuppV^i^	Th3	Th5	Th6	Th6	Th5	Th4	Th5	Th6	Th4	Th4
TlowV	Th11	Th11	Th12	Th12	L1	L1	Th11	L3	Th11	Th11
ThoNM^j^	2	1	3	1	2	1	1	2	1	1
T apex^k^	Th7	Th7	Th9	Th9	Th10	Th10	Th9	Th10	Th7	Th8
LuppV	Th9	Th10	Th12	Th12	Th12	Th12	Th10	Th12	Th10	Th10
LlowV	L4	L4	L4	L4	L4	L5	L4	L4	L4	L4
LumNM	3	2	3	1	2	1	1	1	3	1
L apex	L1	L1	L2	L2	L2	L2	L1	L2	L2	L1
Pt ID^h^	Pre Pt6	Post Pt6	Pre Pt7	Post Pt7	Pre Pt8	Post Pt8	Pre Pt9	Post Pt9		*P* value^l^
TuppV	Th1	Th1	Th7	Th4	Th4	Th5	Th5	Th5		
TlowV	L3	L4	Th12	Th12	Th12	Th12	Th11	Th12		0.508
ThoNM	1	1	2	2	2	1	2	1		0.201
T apex	Th9	Th10	Th9	Th9	Th7	Th8	Th8	Th8		1.00
LuppV	Th11	Th12	Th12	Th12	Th11	Th11	Th11	Th11		
LlowV	L4	L5	L4	L4	L4	L5	L4	L4		0.180
LumNM	1	1	1	1	2	1	3	2		0.023***
L apex	L2	L3	L2	L2	L2	L2	L1	L2		1.00

^a^Pre- and post-injection values for patient ID

^b^Significance level after Wilcoxon signed-rank test (**significant at a level < 0.05)

^c^Pre- and post-injection thoracic Cobb’s angle

^d^Pre- and post-injection lumbar Cobb’s angle

^e^Pre- and post-injection rib vertebrae angle on the concave side

^f^Pre- and post-injection rib vertebrae angle on the convex side

^g^Pre- and post-injection rib vertebrae angle difference

^h^Pre- and post-injection values for patient ID

^i^Upper and lower levels for measurement of Cobbs angle (*T* thoracic, *L* lumbar)

^j^Measurements of Nash and Moe’s classification (*Tho* thoracic, *Lum* lumbar)

^k^Apex vertebra (*T* thoracic, *L* lumbar)

^l^Significance level after Wilcoxon signed-rank test or binomial test (***significant at a level < 0.05)

Table 3 shows the clinical history of using brace treatment and subsequent surgery, the ITB and the clinical feedback throughout the study, where we noted remarks from patients and parents after ITB by open questioning. Two patients reported temporary soreness at the injection site, which regressed within days, and no other serious adverse events occurred during the study, except for one patient who was injected in the erector spine and quadratus lumborum as well as in the PM. No other major medical or orthopedic surgical events at the time of and after termination of the study; the subsequent spinal surgeries took placed years after injection treatment.

**Table 3:** Clinical treatment and Experimental injections

*Pt.* ^*1^	1	2	3	4	5	6	7	8	9
Brace^*2^	*Prov*	*Prov*	*Prov*	*0*	*Prov*	*0*	*Prov*	*Prov*	*Prov*
IniBra^*3^	*7.6*	*6.3*	*3.0*		*6*		*26.5*	*16.4*	*9.3*
*T*ermBra^*4^	*52.4*	*21.7* ^*5^	*51.8*		*28* ^*6^		*67.9*	*42.7*	*31.7*
*TInj* ^*7^	*10.7*	*2.87*	*13.45*	*11.94*	*1*	*10.4*	*51.2*	*5.9*	*13.37*
*IDose* ^*8^	*100*	*100*	*100*	*100*	*100*	*100*	*100*	*100*	*420*
*TMusc* ^*9^	*IP dex*	*IP dex*	*IP dex*	*IP dex*	*IP dex*	*IP dex*	*IP dex*	*IP dex*	*IPQE*
*Med. E* ^*10^	*S, TBP*	*–*	*–*	*–*	*–*	*NBP*	*–*	*–*	*S, NBP*
*Surg. E*	*–*	*SU* ^*13^	*SU* ^*13^	*SU* ^*13^	*cancelled SU* ^*12^	*SU* ^*14^	*–*	*–*	*–*

*1 Patient ID *2 Type of Brace (Prov = Providence brace, 0 = no brace) *3 treated with Brace from time of diagnosis - initiation (months) *4 treated with brace from time of diagnosis - termination (months) *5 Brace abandoned due to physiological disorder of schizophrenia *6 omit brace treatment due to discomfort and psychological reasons *7 treated with injection from time of diagnosis (months) *8 Injection dose (Allergan units) *9 Injection in target muscles (IP = Iliopsoas, IPQE = Iliopsoas, quadratus lumborum and erector spinae, dex = right side, sin = left side) *10 clinical effects or adverse events after injection (S = soreness, NBP = no effect on back pain, TBP = temporary effect on back pain) * 11 surgical history at a later stage (SU = Spinal corrective surgery) *12 Su cancelled due to eating disorder and hormone treatment *13 correction at level Th4-L1 ad modem K2 M *14 correction at level Th3-L2 ad modem MESA Range + removal of cyst in medulla

## Discussion

The temporary muscular paralysis of the PM leads to radiological changes in the spinal deformity of thoracolumbar AIS. These radiographic changes were a significant improvement (lesser curve) in thoracic and lumbar Cobb’s angle and a non-significant thoracic and significant lumbar derotation (changes in Nash and Moe’s classification), and a non-significant small average change in rib vertebra angles with an improvement on the convex side and a deterioration on the concave side. These changes were as expected better in the lumbar region, since the primary effect is in the lumbar region, thus having subsequent less change in the thoracic region as seen in Fig. 2. This implies that the spine muscles do play a role in maintaining the human adolescent idiopathic scoliosis by the muscle contraction or pull by the PM, which was to be expected if the muscle pull by contraction was released in the lumbar area with subsequent effect in the thoracic area as hypnotized earlier. We prescribed the radiological changes to the induced muscular paralysis due to the short follow-up of 6 weeks, since all prior treatments were maintained and no other clinical events occurred in the patient’s life.

A methodological obstacle of this study was to find an adequate way of evaluating our radiographic results. In clinical practice, a Cobb’s angle of at least in between 5° and 10° would be a cutoff value of clinical radiographic change [21]. The diurnal variation in Cobb’s angle for AIS is 5° and the inter- and intra-observer variations are 7.2° and 4.9°, respectively [22]. In this study, we would expect subtle smaller radiographic changes, due to ITB which induces only partial reduction of muscle function [23, 24], and seen in this perspective, we would not expect to detect radiological changes as high as clinical cutoff values [18]. Moreover, three patients had main thoracic curves (patients 3, 4, and 7), and we would expect lesser effect (as in fact seen) than if all patients had main lumbar curves. Additionally, our intra-observer variation for Cobb’s angle was high (average SD of 9.1°) in spite of trying to minimize measuring error by using three blinded experienced doctors and achieve higher accuracy in our radiological recordings by a standardized standing radiographic protocol. For these reasons, we used nonparametric statistical analyses of Wilcoxon signed-rank and one sample binomial test, in which the clinical cutoff value was not included.

In this study, the role of the PM muscle in humans would be scoliogenic, which maintains AIS, but this conclusion can probably not be extrapolated to all of the paravertebral muscles in general. However, to our knowledge, this is the first study in the paravertebral muscles that are influenced directly by the immediate temporary paralysis in humans in order to examine the role in AIS, which in our view is being an important step for the further exploration and understanding of the etiology of AIS. We would recommend to examine this by stimulation instead of paralysis for future studies as suggested by Grivas et al. [10]. Our above-described radiographic changes may be seen as mimicking a “wavy pattern” as described earlier [4], where slight changes in level and size occurred as a response to the almost immediate paralysis of the PM muscle. However, if bilateral paralyses were performed instead of unilateral, this might have resulted in larger changes and have shed light on the role of the paravertebral muscles even further, but bilateral paralysis was omitted for safety reasons to minimize botulinum toxin dosage for the patients to prevent systemic spread, and ethical approval was only for unilateral treatment. Moreover, studies using electromyography and/or magnetic resonance imaging for muscle volume and muscle quality (fatty infiltration) indicate that the spinal muscles are significantly stronger and larger on the convex side at the apex of the curve of the scoliosis [9, 11, 25, 26]; this would indicate that ITB of the paravertebral muscles would have a correcting effect, when injected on the convex side. At the initiation of this study, we evaluated that the muscle contraction/pull of PM on the concave side of the lumbar curve of the thoracolumbar scoliosis in fact brought about the deformity as seen in Fig. 1. In retrospect, the ITB should have been performed either on the convex side or bilaterally, and this could be undertaken in a future study, if another study using ITB in humans was to be undertaken. Our suggestion would be to focus on primary lumbar curves, since the radiological effects were more pronounced in this region. Also, the multifidi and quadratus lumborum muscles have been examined as potential scoliogenic muscles and could be of interest for future studies [10, 27].

The ethical motivation to perform such a study in humans should be discussed. Firstly, our primary ethical motivation for initiating this experimental study was to discover a potential effective corrective or clinical beneficial treatment for AIS. This should be performed strenuously protocolled experimental and monitored study as in this study. The window for effective ITB treatment for AIS would be in the small curve AIS as a supplement for our current conservative treatment of bracing. This might have been able to alleviate humans with brace-treated AIS, since it currently is strenuous and with low compliance to follow [28–30]. From this point of view, it would seem inappropriate not to look for alternate treatment strategies and it certainly would be attractive to find an alternative treatment or to supplement the current conservative treatment. This was our motivation for initiating this study, namely, to investigate if using ITB to treat AIS would lead to improvement of curve and stop curve progression for affected humans. This radiological corrective effect was plausible since we supposedly addressed the culprit of the potential pathology, namely, the PM muscle of the back. However, we did not find radiological corrective effect or patient-reported benefits to a convincing clinical level in our population of patients with AIS—even though radiological correction of significant magnitudes was achieved. We expected that it would be less stressful for the patients when wearing a corrective brace, but we did not find such an effect. In the aftermath of the study, five patients were candidates for surgery, which would suggest that the long-term effect of ITB was not seen. The ITB was in our evaluation unrelated to surgery, since these were performed several years later and ITB have an expected effect of 3 months and severe deterioration after ITB was not seen. However, since we were unable to detect a coherent clinical or corrective treatment for short-term effects, we decided not to perform a second injection in any of the patients in an “interim” analysis after inclusion after the ninth patient.

## Conclusions

In conclusion, this study demonstrated that the paravertebral muscle psoas major do play a role in the pathology in maintaining adolescent idiopathic scoliosis, and this role is maintaining the curvature of the lumbar spine primarily and affecting the curvature of the thoracic spine secondarily.

## Abbreviations

AIS: Adolescent idiopathic scoliosis
ITB: Botulinum toxin A as localized injection therapy
